# RNAi-mediated knockdown of *MTNR1B* without disrupting the effects of melatonin on apoptosis and cell cycle in bovine granulose cells

**DOI:** 10.7717/peerj.4463

**Published:** 2018-04-23

**Authors:** Wenju Liu, Shujuan Wang, Jinxing Zhou, Xunsheng Pang, Like Wang

**Affiliations:** 1 College of Animal Science, Anhui Science and Technology University, Fengyang, Anhui, China; 2 Cell and Molecular Biology Research Center, Anhui Science and Technology University, Fengyang, AnHui, China

**Keywords:** Apoptosis, Granulosa cell, Melatonin, MTNR1B, RNA interference

## Abstract

Melatonin is well known as a powerful free radical scavenger and exhibits the ability to prevent cell apoptosis. In the present study, we investigated the role of melatonin and its receptor *MTNR1B* in regulating the function of bovine granulosa cells (GCs) and hypothesized the involvement of *MTNR1B* in mediating the effect of melatonin on GCs. Our results showed that *MTNR1B* knockdown significantly promoted GCs apoptosis but did not affect the cell cycle. These results were further verified by increasing the expression of pro-apoptosis genes (*BAX* and *CASP3*), decreasing expression of the anti-apoptosis genes (*BCL2* and *BCL-XL*) and anti-oxidant genes (*SOD1* and *GPX4*) without affecting cell cycle factors (*CCND1*, *CCNE1* and *CDKN1A*) and *TP53*. In addition, *MTNR1B* knockdown did not disrupt the effects of melatonin in suppressing the GCs apoptosis or blocking the cell cycle. Moreover, *MTNR1B* knockdown did not affect the role of melatonin in increasing *BCL2*, *BCL-XL*, and *CDKN1A* expression, or decreasing *BAX*, *CASP3*, *TP53*, *CCND1* and *CCNE1* expression. The expression of *MTNR1A* was upregulated after *MTNR1B* knockdown, and melatonin promoted *MTNR1A* expression with or without *MTNR1B* knockdown. However, despite melatonin supplementation, the expression of *SOD1* and *GPX4* was still suppressed after *MTNR1B* knockdown. In conclusion, these findings indicate that melatonin and *MTNR1B* are involved in *BCL2* family and *CASP3*-dependent apoptotic pathways in bovine GCs. MTNR1A and MTNR1B may coordinate the work of medicating the appropriate melatonin responses to GCs.

## Introduction

Ovarian follicle development and the process of ovulation are very complicated processes, which are regulated by various endocrine, paracrine, and autocrine factors. However, less than 1% of follicles reach the preovulatory stage, and the vast majority of them undergo atresia during the follicle development (Kaipia & Hsueh, 1997). Oocytes are surrounded by the granulosa cells (GCs) and numerous researches have focused on the follicular growth and atresia caused by the GCs (Tilly et al., 1991; Jiang et al., 2003; Choi et al., 2011). GCs secrete multiple factors, including gonadal steroids, growth factors, and cytokines, all of which are shown to be essential for their survival and eventual follicular growth (Matsuda et al., 2012; Wang et al., 2017a). Moreover, the apoptosis of GCs could induce the initiator of follicular atresia (Jiang et al., 2003; Choi et al., 2011). Furthermore, GCs can affect the maturation of oocytes through instructive paracrine and junctional interaction (Li & Albertini, 2013). Therefore, follicle development or atresia are mainly dependent on the survival or death of GCs.

Reactive oxygen species (ROS) has been demonstrated to cause oxidative stress and involved in the cell apoptosis. However, under physiological conditions, ROS benefits follicle development, oocyte maturation, and ovulation (Agarwal, Gupta & Sharma, 2005; Sena & Chandel, 2012). On the contrary, excessive ROS induces GCs apoptosis, results in antral follicle atresia and affects the oocyte quality (Gupta et al., 2006; He et al., 2016d). Therefore, the balance between formation and elimination of ROS is crucial for GCs survival and development. Similarly, balancing between pro-apoptotic and anti-apoptotic factors are crucial such as the BCL-2 family (BCL2, BCL-XL, and BAX), CASP3 (CASPASE-3), and TP53 (Tumor protein p53) play important roles in modulating the GCs apoptosis through up- or down-regulating their expression.

Melatonin is mainly secreted by pineal gland and exerts its physiological function primarily through binding to its receptors. Among them, MTNR1A and MTNR1B (also known as MT1 and MT2, respectively) are high-affinity G protein-coupled receptors (Dubocovich & Markowska, 2005). Recent research demonstrates that ovaries have the ability to secrete the melatonin (Reiter et al., 2014). Moreover, the follicular fluid have a higher concentration of melatonin and its concentration increase with increasing follicular diameter (Tamura et al., 2013). Recently, it has been demonstrated that melatonin could directly modulate ovarian function. For example, melatonin is involved in folliculogenesis, follicle selection, oocyte maturation, protecting GCs from ROS, as well as regulating the GCs secretion (Dubocovich & Markowska, 2005; Tamura et al., 2009, 2013; Wang et al., 2012a, 2017a). Our previous research showed that exogenous melatonin significantly suppressed GCs apoptosis (Wang et al., 2012a). Melatonin protects the integrity of GCs and decreases the GCs apoptosis by reducing oxidative stress in nuclei, mitochondria, and plasma membranes in mice (Tanabe et al., 2015). Melatonin could prevent the apoptosis of porcine GCs during follicular atresia through its free-radical-scavenging activity (He et al., 2016a). While apoptosis-related factors, such as BCL2 family, TP53, CASP3, and ROS also are involved in the cell apoptosis progress upon apoptosis induction, and melatonin could modulate their response (Fu et al., 2014; Cruz et al., 2014a; He et al., 2016b). MTNR1B is involved in the phase-shifting response and entrainment of circadian rhythms (Dubocovich et al., 1998; Dubocovich & Markowska, 2005), and modulates immune and inflammatory responses (Drazen & Nelson, 2001; Drazen et al., 2001). However, the role of the MTNR1B is little known in the GCs. Therefore, in this study, we assessed the role of MTNR1B in regulating cellular progression, apoptosis, and expression of anti-oxidants genes in bovine GCs.

Melatonin is critical for protecting the integrity and function of GCs. However, the mechanism by which melatonin affects the apoptosis and function of GCs, especially the melatonin receptors mediating the beneficial effects of melatonin on GCs, is still unclear. In the current study, we investigated the role of *MTNR1B* in regulating cell cycle and apoptosis of bovine GCs by RNAi of *MTNR1B*. Moreover, we further revealed whether *MTNR1B* knockdown affected the GCs response to melatonin by assessing the expression of apoptosis-related genes (*BCL2*, *BCL-XL*, *TP53*, *BAX*, and *CASP3*), anti-oxidants genes (*GPX4* [Glucose peroxidase 4] and *SOD1* [Superoxide dismutase 1]), and cell cycle factors (*CCND1* [*Cyclin D1*], *CCNE1* [*Cyclin E1*], and *CDKN1A* [*P21*]). The present study would be better the understanding of melatonin and MTNR1B local regulation within bovine GCs.

## Materials and Methods

### Chemicals

The antibodies including CASP3 rabbit polyclonal antibody (ab90437) and anti-MTNR1B rabbit polyclonal antibody (ab203346) were purchased from Abcam, Cambridge, MA, USA. All the other antibodies including MTNR1A goat monoclonal antibody (SC-13186), BAX rabbit polyclonal antibody (SC-526), TP53 mouse monoclonal antibody (SC-99), BCL2 rabbit polyclonal antibody (SC-492), ACTB (actin beta) mouse monoclonal antibody (SC-47778), goat anti-rabbit lgG-HRP(SC-2054), chicken anti-goat lgG-HRP (SC-2961), and goat anti-mouse lgG-HRP (SC-2005) were purchased from the Santa Cruz Biotechnology, Inc., Dallas, TX, USA.

Melatonin was purchased from Sigma-Aldrich (St. Louis, MO, USA), dissolved in ethanol at the concentration of 100 μg/ml, and then diluted to the final concentration (1,200 pg/ml) with Dulbecco’s modified essential medium (DMEM) (Gibco, Grand Island, NY, USA) before adding to the cultured GCs.

### Bovine GCs isolation and culture

Granulosa cells collection was performed as previously described elsewhere (Wang et al., 2012a, 2017b). A total of 50–60 bovine ovaries were obtained from Bengbu abattoir (Anhui, China), washed three times by 70% alcohol and sterile 0.9% NaCl. GCs were isolated from 3 to 6 mm antral follicles using a syringe and sterile needle puncture method. The cell pellets were digested for 5 min using 0.25% trypsin with 0.025% EDTA (Gibco, Grand Island, NY, USA). GCs were collected by centrifugation (1,500 rpm, 5 min) and were cultured in DMEM (Gibco, Grand Island, NY, USA) supplemented with streptomycin (50 μg/ml), penicillin (50 IU/ml) (Pen-Strep, Invitrogen, Carlsbad, CA, USA), plasmocin (25 μg/ml; InvivoGen, San Diego, CA, USA), and 10% fetal bovine serum (FBS; Hyclone, South Logan, UT, USA) at 37 °C in an incubator containing 5% CO_2_. In this study, the protocols for the experiment were reviewed and approved by the Institutional Committee on Animal Care and Use at Anhui Science and Technology University, and experiments were repeated three times independently.

### Construction and transfection of recombinant pSIREN-RetroQ-ZsGreen Vectors

The coding sequence of bovine *MTNR1B* (NM 001206907) was derived from the NCBI GenBank database (https://www.ncbi.nlm.nih.gov/gene/4544). Three siRNA target sites were selected according to the siRNA online program (http://rnaidesigner.thermofisher.com) (Table 1). The typical structure of the short hairpin RNA contains a restriction site at its 5′ end, a 19-base sense strand, nine bases for the hairpin loop, a 19-base antisense strand, six bases for the terminator, and six bases corresponding to a unique *Hind*III restriction site (allowing for identification of the recombinant plasmids by restriction enzyme digestion) as described elsewhere Han et al. (2013) and Wang et al. (2017a). These oligonucleotides were annealed and inserted into the *Bam*HI and *Eco*RI sites of the RNAi-Ready pSIREN-RetroQ-ZsGreen Vector (BD Biosciences, San Jose, CA, USA). The recombinant plasmids were named as pshRNA-1, pshRNA-2, and pshRNA-3, respectively (Table 1). The pshRNA-negative with a nonsense sequence was used as a negative control. In addition, pSIREN-RetroQ-ZsGreen Vector independently expressed a green fluorescent protein (GFP). which could be used to monitor the delivery efficiency of transfection construct using fluorescence microscopy. All plasmids were extracted using EndoFree Maxi Plasmid Kit (Tiangen, Beijing, China) and confirmed by sequencing.

**Table 1:** Target sequences of bovine *MTNR1B*.

Name	Target sequence(5′ → 3′)	Position on cds
pshRNA-1	GGAACGCAGGTAACCTGTTCT	215
pshRNA-2	GCTACTTCCTGGCCTATTTCA	878
pshRNA-3	GGGAATACAAGAGGATCATC	950
pshRNA-negative	CTTCATAAGGCGCATAGC	

One day before transfection, 2–5 × 10^5^ cells were cultured in a 12-well plate to reach 70–80% confluence at the time of transfection. GCs were transfected with pshRNA-1, 2, 3, or pshRNA-negative using Lipofectamine^TM^ LTX with Plus^TM^ Reagent (Invitrogen, Carlsbad, CA, USA) according to manufacturer’s instructions. Transfection medium was changed after 6 h to fresh growth medium without antibiotics. The expression of GFP was observed under a fluorescence microscope to examine transfection efficiency beginning from 24 h after transfection. GCs were collected for RNA, protein extraction, and other experiments in respective time intervals.

### RNA extraction and real-time PCR

Total RNA was isolated using RNAprep pure cell Kit (Tiangen, Beijing China), and treated with RNase-free DNaseI for the removal of genomic DNA. The total RNA was reverse-transcribed to cDNA with a RevertAid First Strand cDNA Synthesis Kit (ThermoFisher Scientific, Waltham, MA, USA) according to the manufacturer’s instructions. The quantitative real-time PCR was performed using LightCycler 480 II Real-Time PCR System (Roche, Mannheim, Germany). The amplification reaction was carried out with LightCycler 480 SYBR Green I Master Mix (Roche, Penzberg, Germany). The specific primer pairs were listed in Table 2. To exclude nonspecific PCR product, a melting curve analysis was performed after real-time PCR reactions. The expression levels of the target genes were normalized to *ACTB* in each sample. The related mRNA expression levels were estimated using the 2^−ΔΔ*C*T^ method (Livak & Schmittgen, 2001).

**Table 2:** Sequences of primer pairs for quantitative real-time PCR.

Gene	Forward primer sequence (5′ → 3′)	Reverse primer sequence (5′ → 3′)	length
*BAX*	TGCAGAGGATGATCGCAGCTGTG	CCAATGTCCAGCCCATCATGGTC	198
*BCL2*	CGCATCGTGGCCTTCTTTGAGTT	GCCGGTTCAGGTACTCAGTCAT	115
*BCL-XL*	ATGGCAGCAGTAAAGCAAG	GCTGCATTGTTCCCATAGA	236
*CASP3*	*CAGACAGTGGTGCTGAGGATGA*	*GCTACCTTTCGGTTAACCCGA*	211
*TP53*	CCTCCCAGAAGACCTACCCT	CTCCGTCATGTGCTCCAACT	221
*GPX4*	TGTGCTCGCTCCATGCACGA	CCTGGCTCCTGCCTCCCAA	224
*SOD1*	GCTGTACCAGTGCAGGTCCTCA	CATTTCCACCTCTGCCCAAGTC	228
*CCND1*	GCCCTCGGTGTCCTACTTCAA	ACAGGAAGCGGTCCAGGTAGT	152
*CCNE1*	CCTCCAAAGTTGCACCAGTT	AGGATACTGAGGCAGGAGCA	195
*CDKN1A*	CGTCTCAGGAGGACCACTT	TCAGTCTGCGTTTGGAGTG	159
*MTNR1A*	CTGCCACAGCCTCAGATACA	GAGCATCGGAACGATGAAAT	217
*MTNR1B*	GGAGCTTTCTGAGCATGTTTG	CCCTGCGGAAGTTCTGGTT	210
*ACTB*	CATCGGCAATGAGCGGTTCC	CCGTGTTGGCGTAGAGGTCC	145

### Western blot analysis

The bovine GCs were harvested at 48 h, washed with cold PBS and lyzed in RIPA buffer (Thermo Pierce, Rochford, IL, USA) containing protease inhibitor cocktail (Sigma, St. Louis, MO, USA). After 0.5 h incubation at 4 °C, GCs were centrifuged at 12,000*g* for 15 min. Total protein concentrations were ascertained using BCA-assay (Thermo Pierce, Rochford, IL, USA), then denatured at 100 °C for 5 min in SDS loading buffer and frozen at −80 °C until use. Approximately, 30 μg of protein samples were separated by 12% polyacrylamide gel. The gels were then transferred to a polyvinylidene fluoride membrane (Millipore, Bedford, MA, USA), which were then blocked for 1 h with 5% skim milk (BD Biosciences, San Jose, CA, USA) in Tris-buffered saline (25 mM Tris, pH 7.6 and 150 mM NaCl) containing 0.1% Tween 20 (Sigma-Aldrich, St. Louis, MO, USA) (TBST), and then incubated overnight at 4 °C with following primary antibody: anti-MTNR1B (1:400, Abcam, Cambridge, MA, USA), anti-CASP3 (1:500, Abcam, Cambridge, MA, USA), anti-MTNR1A (1:400, Santa Cruz Biotechnology, Inc., Dallas, TX, USA), anti-BCL2 (1:500, Santa Cruz Biotechnology, Inc., Dallas, TX, USA), anti-TP53 (1:500, Santa Cruz Biotechnology, Inc., Dallas, TX, USA), anti-BAX (1:500, Santa Cruz Biotechnology, Inc., Dallas, TX, USA), and anti-ACTB (1:1000, Santa Cruz Biotechnology, Inc., Dallas, TX, USA). Next, membranes were washed three times with TBST, and immediately incubated for 1 h at 37 °C with HRP labeled chicken anti-goat, goat anti-rabbit or goat anti-mouse secondary antibodies (1:5,000; Santa Cruz Biotechnology, Inc., Dallas, TX, USA) and washed three times with TBST. Finally, bands were visualized using clarity western ECL substrate (Bio-Rad, Hercules, CA, USA) and scanned using a ChemiDoc XRS chemiluminescent imaging system (Bio-Rad, Hercules, CA, USA).

### Cell cycle detection by flow cytometry

Cells were seed into 12-cell plates and harvested 48 h after transfection by digestion with trypsin at 37 °C for 5 min. The cells then were washed with PBS, fixed in cold 70% ethanol at 4 °C overnight, washed again in PBS and then incubated with 50 μg/ml propidium iodide (PI) with 100 μg/ml RNase A (Tiangen, Beijing, China) in dark for 30 min at room temperature. The cells cycle was detected by FACSVerse flow cytometry (Becton, Dickinson and Company, Franklin Lakes, NJ, USA), and the data was analyzed by the ModFit LT for Mac V3.0 software (Verity Software House, Topsham, ME, USA). For each determination, a minimum of 10,000 cells was analyzed. All experiments were performed independently three times.

### Cell apoptosis detection by flow cytometry

Granulosa cells were cultured and harvested at 48 h. The cells were digested by 0.25% trypsin without EDTA (Gibco, Grand Island, NY, USA). Cells apoptosis was detected by Annexin V-APC/7-AAD Apoptosis Detection Kit (Hangzhou Multi Sciences, Co., Hangzhou, China) according to the manufacturer’s instruction. After incubation for 15 min at 37 °C in dark, the stained cells were analyzed by FACSVerse flow cytometry (Becton, Dickinson and Company, Franklin Lakes, NJ, USA). Experiments were repeated three times independently.

### Experiment of design

*MTNR1B* knockdown and melatonin supplementation were used to determine whether the effect of melatonin on GCs was mediated via MTNR1B. Our previous research identified that the GCs apoptosis were significantly suppressed 48 h after melatonin treatment (1,200 pg/ml) (Wang et al., 2012a). To determine the effects of melatonin and MTNR1B on bovine GCs, 1,200 pg/ml melatonin were supplemented into the cultured GCs, when transfection medium was changed into fresh medium. Therefore, the experiment was divided into four experimental groups: (1) pshRNA-negative group (control); (2) pshRNA-2; (3) pshRNA-2 plus melatonin; and (4) melatonin. In addition, the GCs cell cycle and apoptosis were analyzed by flow cytometry in each experiment. Moreover, the expression level of apoptosis related genes (*BCL2*, *BCL-XL*, *BAX*, *CASP3*, and *TP53*), anti-oxidatant related genes (*SOD1* and *GPX4*), and cell cycle factors (*CCND1*, *CCNE1*, and *CDLN1A*) were investigated by real-time PCR in each experiment while the apoptotic related protein levels were tested by Western blot.

### Statistical analysis

All data were presented as Mean ± SEM of triplicate experiments (*n* = 3). Significant difference was evaluated using Duncan’s multiple comparisons following one-way ANOVA with the General Linear Models Procedure of Statistical Analysis Systems (SAS Inc., Cary, NC, USA). *P* < 0.05 was considered significant.

## Results

### Construction and identification the efficiency of *MTNR1B* RNAi recombinant plasmids in bovine GCs

**Figure 1:**
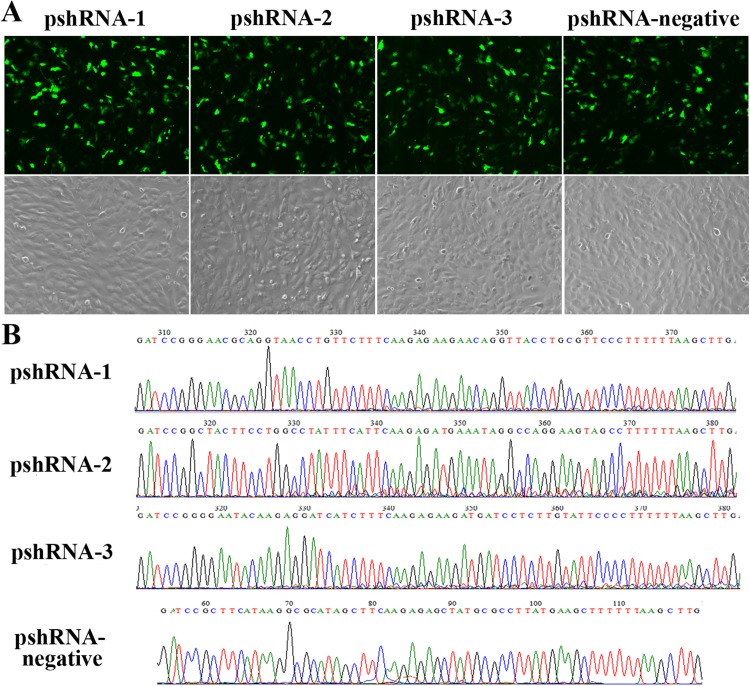
Construction and identification the efficiency of *MTNR1B* RNAi recombinant plasmids in bovine GCs. (A) Transfection efficiency, as reported by green fluorescence, for GCs containing pshRNA-1, pshRNA-2, pshRNA-3, and pshRNA-negative. After 48 h, the expression of GFP were shown in the GCs, which implied that *MTNR1B* RNAi recombinant plasmids were high efficiently expressed. Untransfected GCs were included as a control. (B) Sequencing of plasmids pshRNA-1, -2, -3, and pshRNA-negative. These clones were further confirmed by sequencing. No mutations were found in the inserted hairpin fragments.

**Figure 2:**
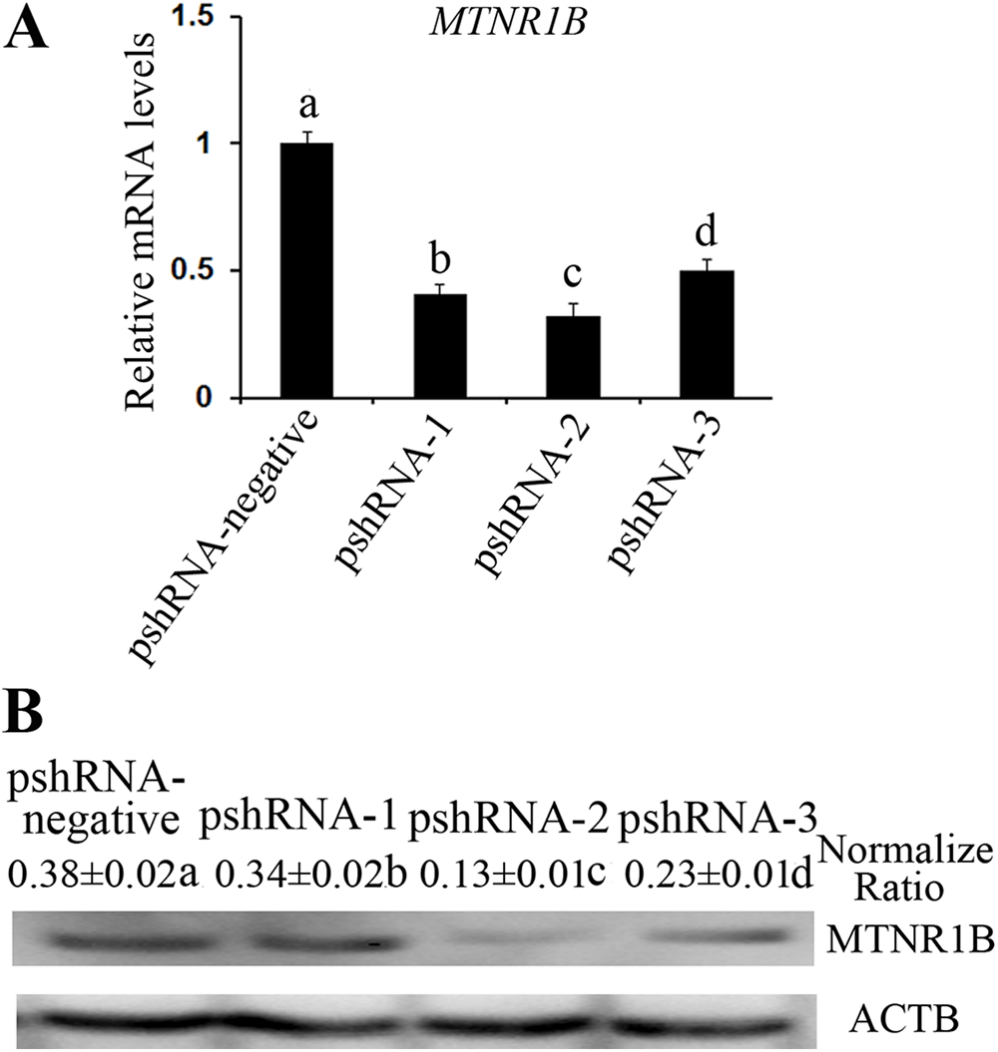
Identification the transfection efficiency of pshRNA-1, pshRNA-2, pshRNA-3, and pshRNA-negative at 48 h after transfection in bovine GCs. (A) *MTNR1B* mRNA level was detected in bovine GCs after 48 h transfected with *MTNR1B* RNAi recombinant plasmids. (B) MTNR1B protein level was detected using western blotting in the GCs transfected with *MTNR1B* RNAi recombinant plasmids, after 48 h. All three plasmids were able to silence the *MTNR1B* mRNA and protein level, with pshRNA-2 having greatest effective compared to other plasmids. The statistical differences were tested using one-way ANOVA. The data with different lowercase letters (a, b, c, and d) were significantly different (*P* < 0.05).

All *MTNR1B* recombinant plasmids were confirmed by sequencing and the oligonucleotides for *MTNR1B* were inserted correctly (Fig. 1A). In addition, these vectors independently expressed a GFP and the efficiency of transfection was confirmed by observing the expression of GFP (Fig. 1B) beginning at 24 h after transfection. Moreover, the silencing efficiency was corroborated through the *MTNR1B* mRNA level by real-time PCR and protein levels by Western blot. The results indicated that all three plasmids were able to silence the *MTNR1B* mRNA and protein level, with pshRNA-2 having greatest effective at downregulating of mRNA (68%) and protein level compared to other plasmids (Fig. 2). Therefore, the pshRNA-2 was selected for further progression of the experiments.

### Effects of *MTNR1B* gene knockdown and melatonin supplementation on the cell cycle

**Figure 3:**
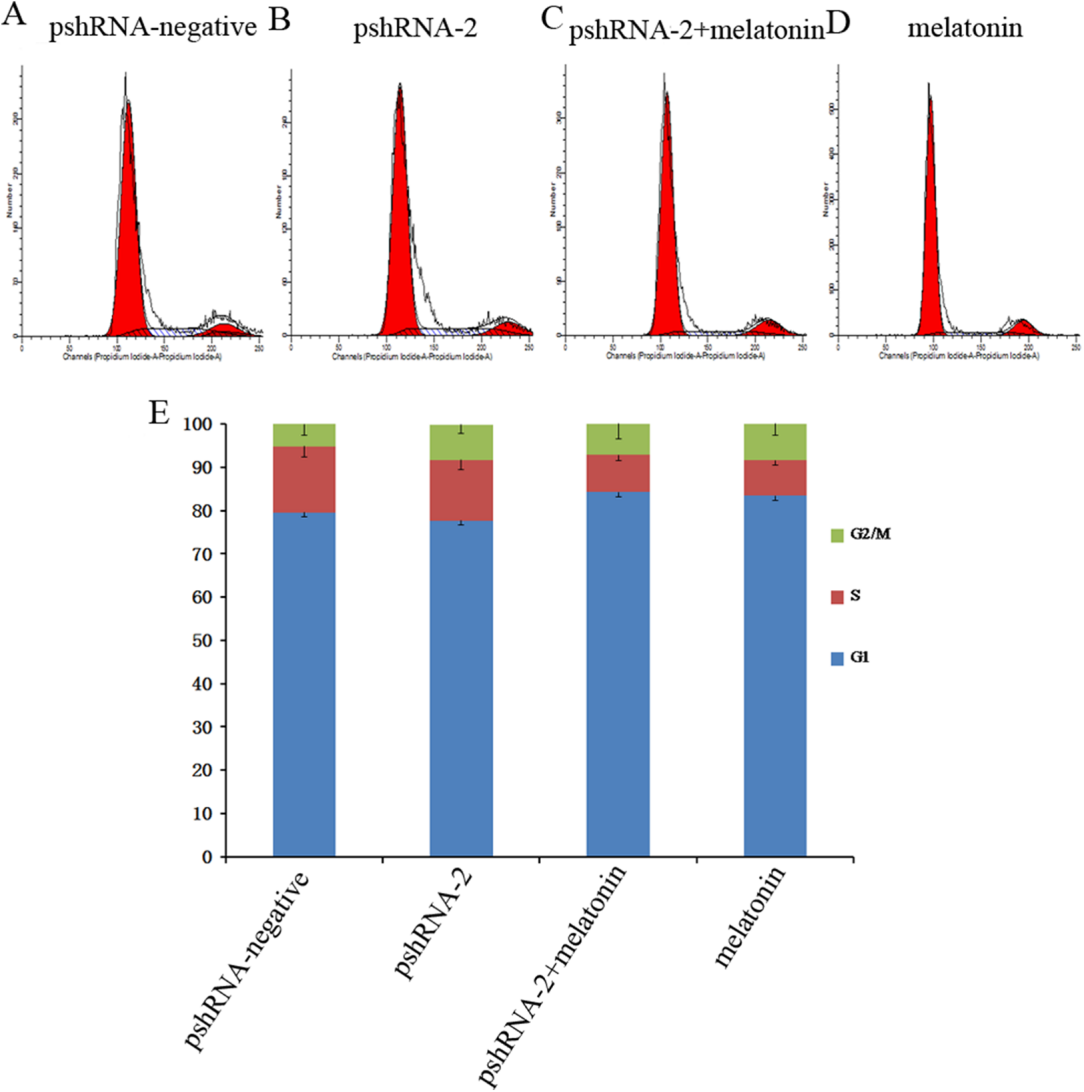
Effects of *MTNR1B* gene silencing and melatonin supplementation on the cell cycle. GCs were transfected with pshRNA-2 and treated with melatonin in the 48 h treatment (1,200 pg/mL). Cell cycle was analyzed by FACSVerse after PI staining. (A–E): *MTNR1B* silencing did not show significant changes in the cell cycle after 48 h transfected with pshRNA-2; after 48 h of treatment, GCs were arrested in G1 phases and showed a decrease in the S phases in the pshRNA-2 + melatonin group and the melatonin group compared with the pshRNA-2 group and the control groups. The results are representative of three independent experiments.

*MTNR1B* knockdown did not significantly alter the GCs population compared to the control group (Fig. 3, *P* > 0.05). Furthermore, melatonin supplementation significantly increased the G1 phase of cell cycle accompanied with the decreased S phase in pshRNA-2 + melatonin group and the melatonin group compared to the pshRNA-2 and the control groups (Fig. 3, *P* < 0.05), and there was no significant difference in the cell cycle between the pshRNA-2 + melatonin group and the melatonin group (Fig. 3, *P* < 0.05). This indicated that melatonin played an important role in the growth progression of bovine GCs and *MTNR1B* knockdown did not affect the effect of melatonin on bovine GCs cycle.

**Figure 4:**
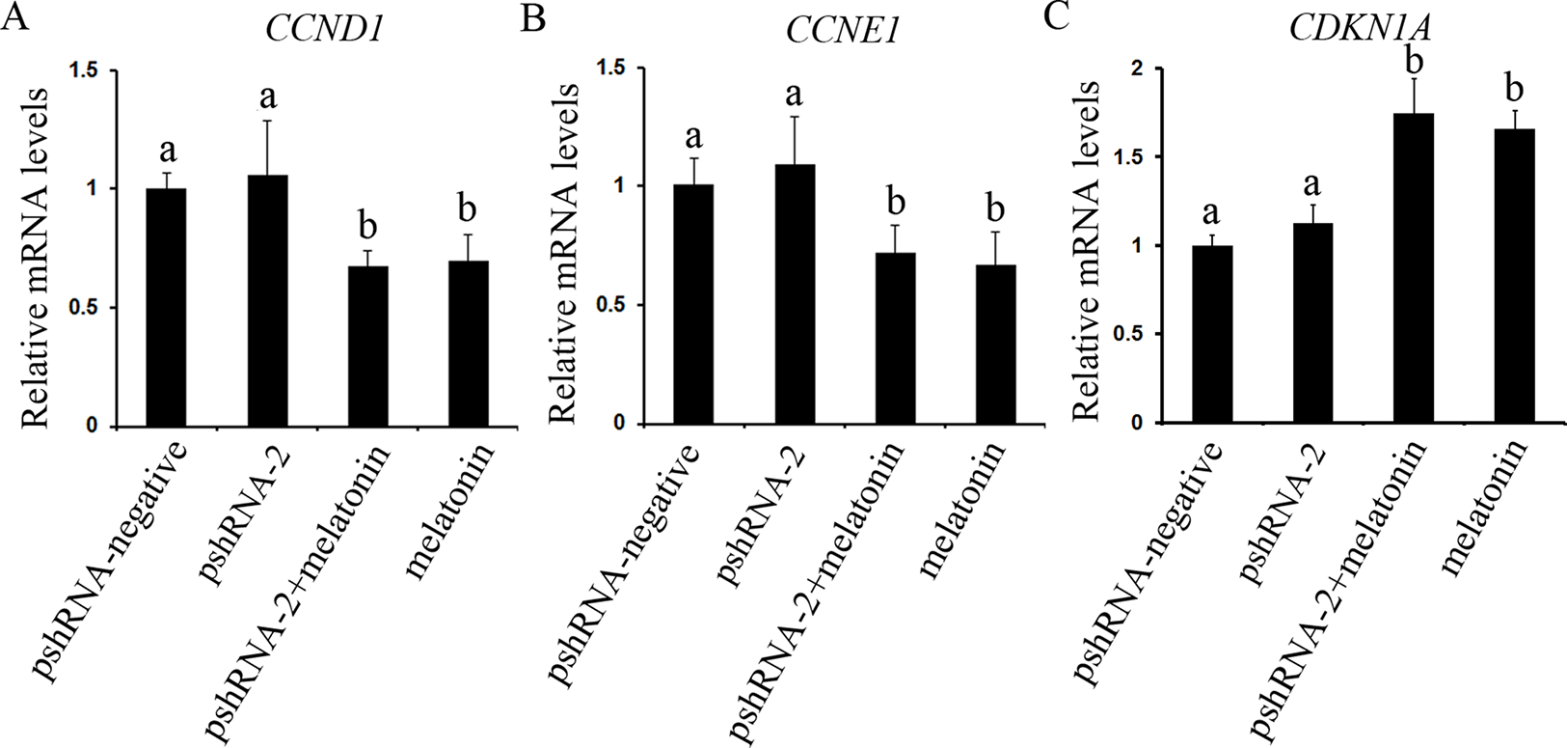
Effects of *MTNR1B* gene silencing and melatonin supplementation on the expression of cell cycle factors (*CCND1*, *CCNE1*, and *CDKN1A*). The mRNA levels of cell cycle factors related genes were examined by real-time PCR in GCs at 48 h after transfection with pshRNA-2 and/or melatonin treatment. (A–C): The expression of *CCND1*, *CCNE1*, and *CDKN1A* were not significantly different in the pshRNA-2 group than that in the control group. However, melatonin significantly upregulated the expression of *CDKN1A* and downregulated the expression of *CCND1* and *CCNE1* with or without *MTNR1B* knockdown compared to the pshRNA-2 group and the control group. The quantity of mRNA was normalized to that of *ACTB*. The data with different lowercase letters (a and b) were significantly different (*P* < 0.05).

The effect of *MTNR1B* knockdown and melatonin supplementation was further elucidated by determining the mRNA expression of cell cycle related genes (*CCND1*, *CCNE1*, and *CDKN1A*) by real-time PCR. *MTNR1B* knockdown did not significantly alter the expression of *CCND1*, *CCNE1*, and *CDKN1A* (Fig. 4, *P* > 0.05). However, melatonin significantly upregulated the expression of *CDKN1A* and downregulated the expression of *CCND1* and *CCNE1* with or without *MTNR1B* knockdown compared to the pshRNA-2 and control groups (Fig. 4, *P* < 0.05). Moreover, *CCND1*, *CCNE1*, and *CDKN1A* expression was not differ between the pshRNA-2 + melatonin group and the melatonin group (Fig. 4, *P* > 0.05). These results demonstrated that melatonin might regulate the cell cycle through upregulating the expression of *CDKN1A* and downregulating the expression of *CCND1* and *CCNE1*, which were not affected by *MTNR1B* knockdown.

### Effects of *MTNR1B* gene knockdown and melatonin supplementation on GCs apoptosis

The effects of *MTNR1B* and melatonin on regulating of apoptosis in GCs were analyzed with Annexin V-APC/7-AAD by FACSVerse flow cytometry. *MTNR1B* silencing significantly increased the apoptotic cells compared to control group (Table 3, *P* < 0.05). In contrast, melatonin significantly inhibited the cells apoptosis with or without *MTNR1B* knockdown compared to that in the pshRNA-2 and control groups (Table 3, *P* < 0.05).

**Table 3:** Analysis of apoptosis with *MTNR1B* silencing and melatonin added in bovine GCs (Mean ± SEM, n = 3).

Groups	Live cells (%)	Apoptotic (%)
**pshRNA-negative**	85.50 ± 0.40a	14.39 ± 0.45a
**pshRNA-2**	80.73 ± 0.45b	19.17 ± 0.44b
**pshRNA-2 + melatonin**	83.73 ± 1.28c	16.17 ± 1.32c
**melatonin**	94.80 ± 0.82d	5.13 ± 0.80d

**Note:**

All results were evaluated by one-way ANOVA. The data with different letters indicates the level of significance in column (*P* < 0.05).

**Figure 5:**
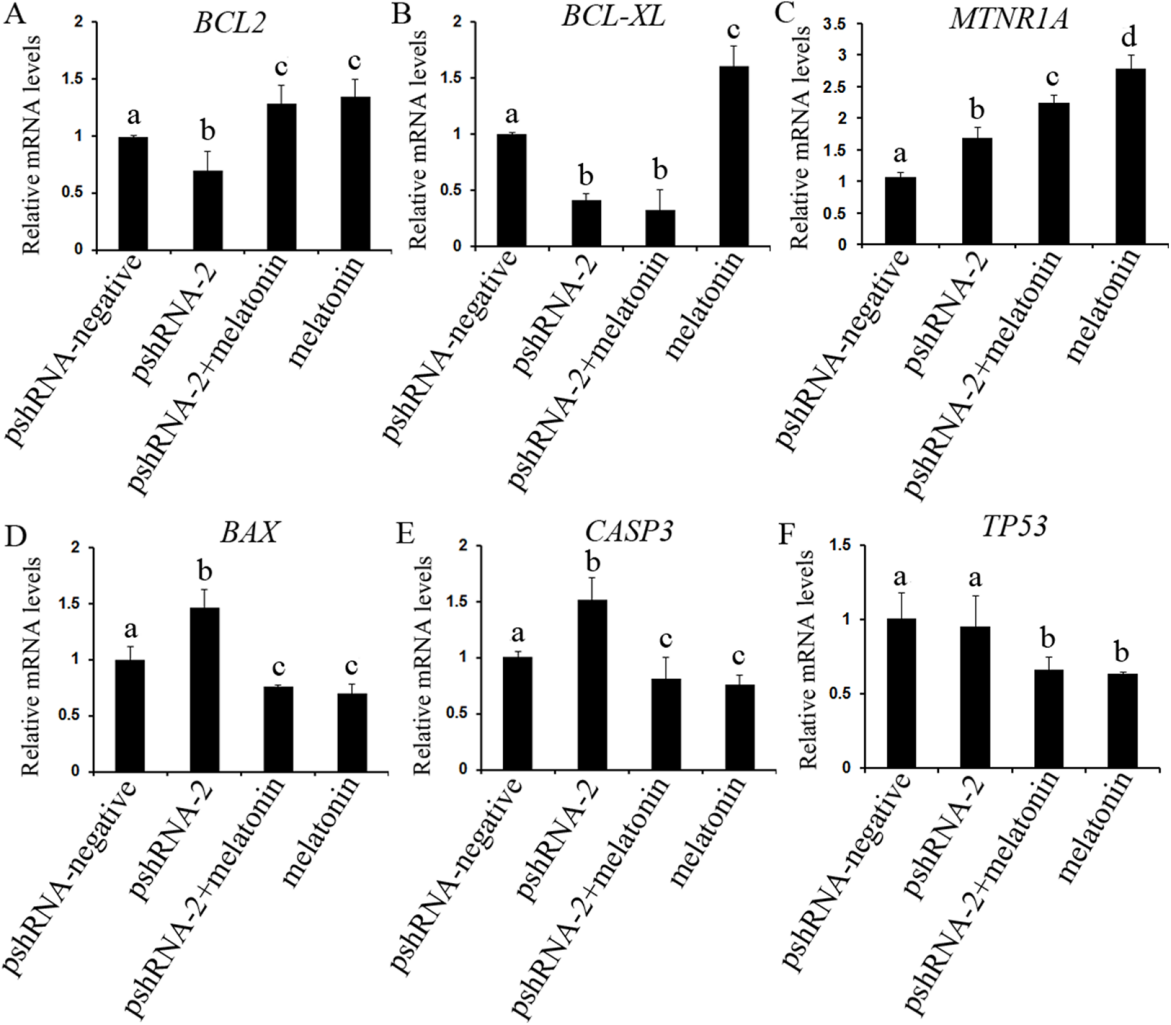
Effect of *MTNR1B* silencing and melatonin supplementation on the expressions of apoptosis related genes (*BCL2*, *BCL-XL*, *TP53*, *BAX*, *CASP3*, and *MTNR1A*). The mRNA levels of apoptosis related genes were examined by real-time PCR in GCs at 48 h after transfection with pshRNA-3 and/or melatonin treatment. (A–F): *MTNR1B* knockdown significantly upregulated the expression of *CASP3*, *BAX*, and *MTNR1A* while downregulating the expression of *BCL2* and *BCL-XL*, without significant difference in the expression of *TP53* compared to control group. Melatonin significantly upregulated the expression of *BCL2* and *MTNR1A* while downregulating the expression of *BAX*, *CASP3*, and *TP53* with or without *MTNR1B* knockdown compared to the pshRNA-2 group and the control group. The quantity of mRNA was normalized to that of *ACTB*. The data with different lowercase letters (a, b, c, and d) were significantly different (*P* < 0.05).

**Figure 6:**
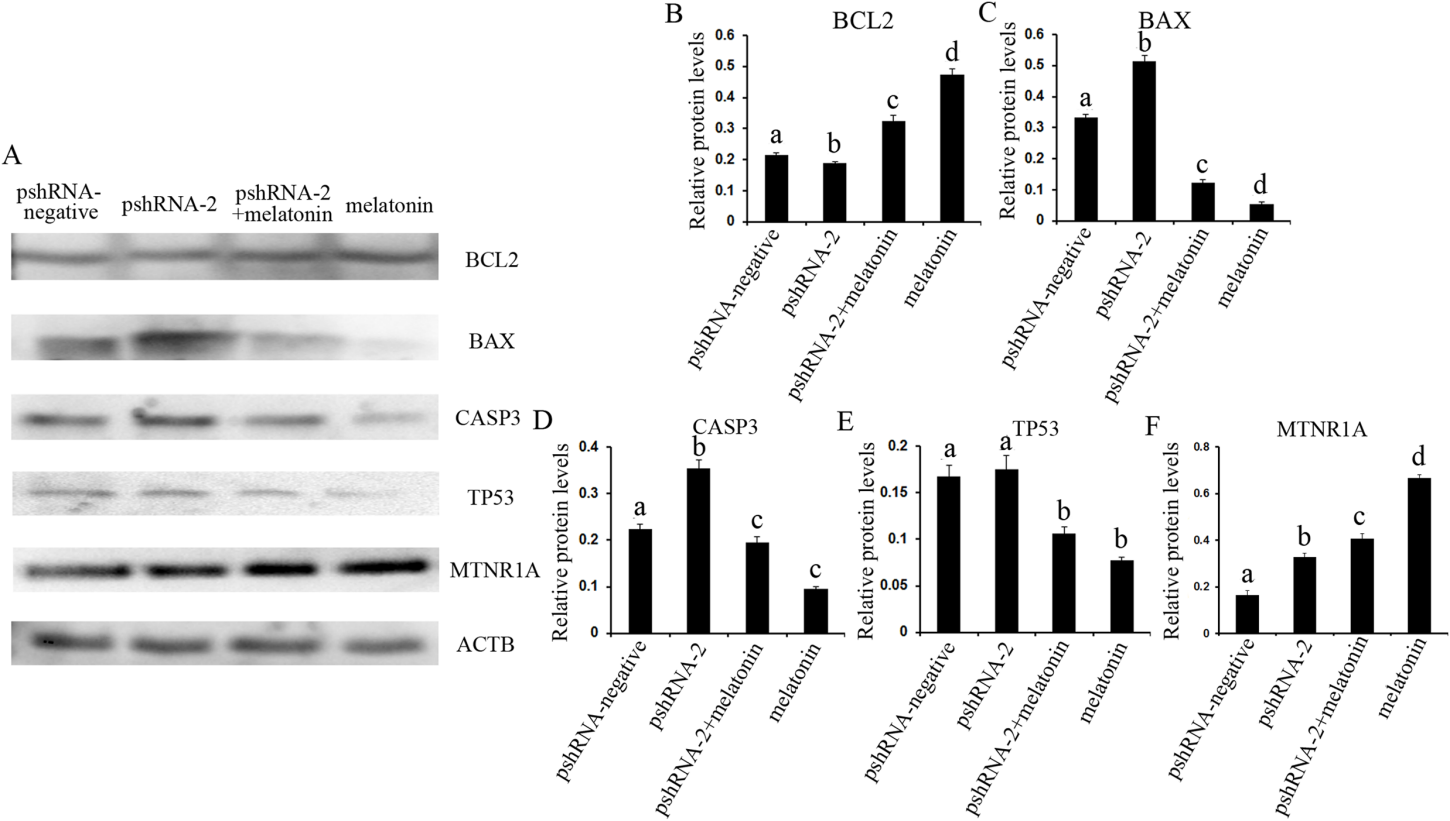
Effect of *MTNR1B* silencing and melatonin supplementation on the expression of apoptosis related protein (BCL2, TP53, BAX, CASP3, and MTNR1A) in GCs. Protein expression level of BCL2, TP53, BAX, and CASP3 was detected by western blot at 48 h after transfection with pshRNA-3 and/or melatonin treatment. (A–F): The expression of CASP3, BAX, and MTNR1A was significantly upregulated, and the expression of BCL2 was downregulated, without significant difference in the expression of TP53 in the pshRNA-2 group than that in the control group. Moreover, the expression of BCL2 and MTNR1A was increased and the expression of BAX, CASP3, and TP53 was decreased in the pshRNA-2 + melatonin group and the melatonin group compared to the pshRNA-2 group and the control group. The normalized ratio for each protein was calculated by dividing the mean signal intensity from three biological replicates by the mean signal intensity with ACTB. The data with different lowercase letters (a, b, c, and d) were significantly different (*P* < 0.05).

The role of MTNR1B and melatonin in regulation of apoptosis was further investigated by assessing the genes expression level of *BCL2* (both mRNA and protein level), *CASP3* (both mRNA and protein level) and *BCL-XL* (mRNA level), *BAX* and *TP53* (both mRNA and protein level) (Figs. 5 and 6). The results showed that *MTNR1B* knockdown significantly upregulated the expression of *CASP3* and *BAX* while downregulating the expression of *BCL2* and *BCL-XL* (*P* < 0.05). However, there was no significant difference in the expression of *TP53* after *MTNR1B* knockdown (*P* > 0.05). These results revealed that *MTNR1B* inhibited GCs apoptosis through *BCL2* family and *CASP3*-dependent apoptotic pathway.

To further reveal whether *MTNR1B* knockdown affect the effects of melatonin on GCs, the expression of *BCL2*, *BCL-XL, CASP3*, *BAX*, and *TP53* were measured after melatonin treatment. Melatonin significantly upregulated the expression of *BCL2* while decreasing the expression of *BAX*, *CASP3*, and *TP53* with or without *MTNR1B* knockdown compared to the pshRNA-2 and control groups (Figs. 5 and 6, *P* < 0.05), and there was no significant difference between the pshRNA-2 + melatonin and the melatonin groups (*P* > 0.05). In addition, the expression of *BCL-XL* was higher in the melatonin group than other groups, however, lower in the pshRNA-2 group and the pshRNA-2 + melatonin group than that in the control group and the melatonin group, and no significant difference in *BCL-XL* expression was observed between the pshRNA-2 group and the pshRNA-2 + melatonin group (*P* > 0.05). These findings suggested that *MTNR1B* knockdown did not affect the effect of melatonin on inhibiting *BAX*, *CASP3*, and *TP53* expression and promoting *BCL2* expression, and *MTNR1B* knockdown could affect the effect of melatonin on promoting *BCL-XL* expression.

### Effects of *MTNR1B* gene knockdown and melatonin supplementation on *MTNR1A* gene expression

The expression of *MTNR1A* was also detected after *MTNR1B* silencing and melatonin supplementation to see if *MTNR1B* knockdown could cause *MTNR1A* compensatory alteration within the GCs. *MTNR1A* expression was increased both after *MTNR1B* silencing and melatonin treatment (*P* < 0.05) (Figs. 5 and 6). However, the expression of *MTNR1A* was significantly increased in the pshRNA-2 + melatonin group compared to the pshRNA-2 group, and was decreased than that in the melatonin group (*P* < 0.05) (Fig. 6). *MTNR1B* silencing did not inhibit melatonin responses to GCs, and the *MTNR1A* compensatory alteration maybe contribute to this. Hence, MTNR1A and MTNR1B may coordinate the work of mediating the appropriate melatonin responses to GCs.

### Effects of *MTNR1B* gene knockdown and melatonin supplementation on anti-oxidant related genes expression

**Figure 7:**
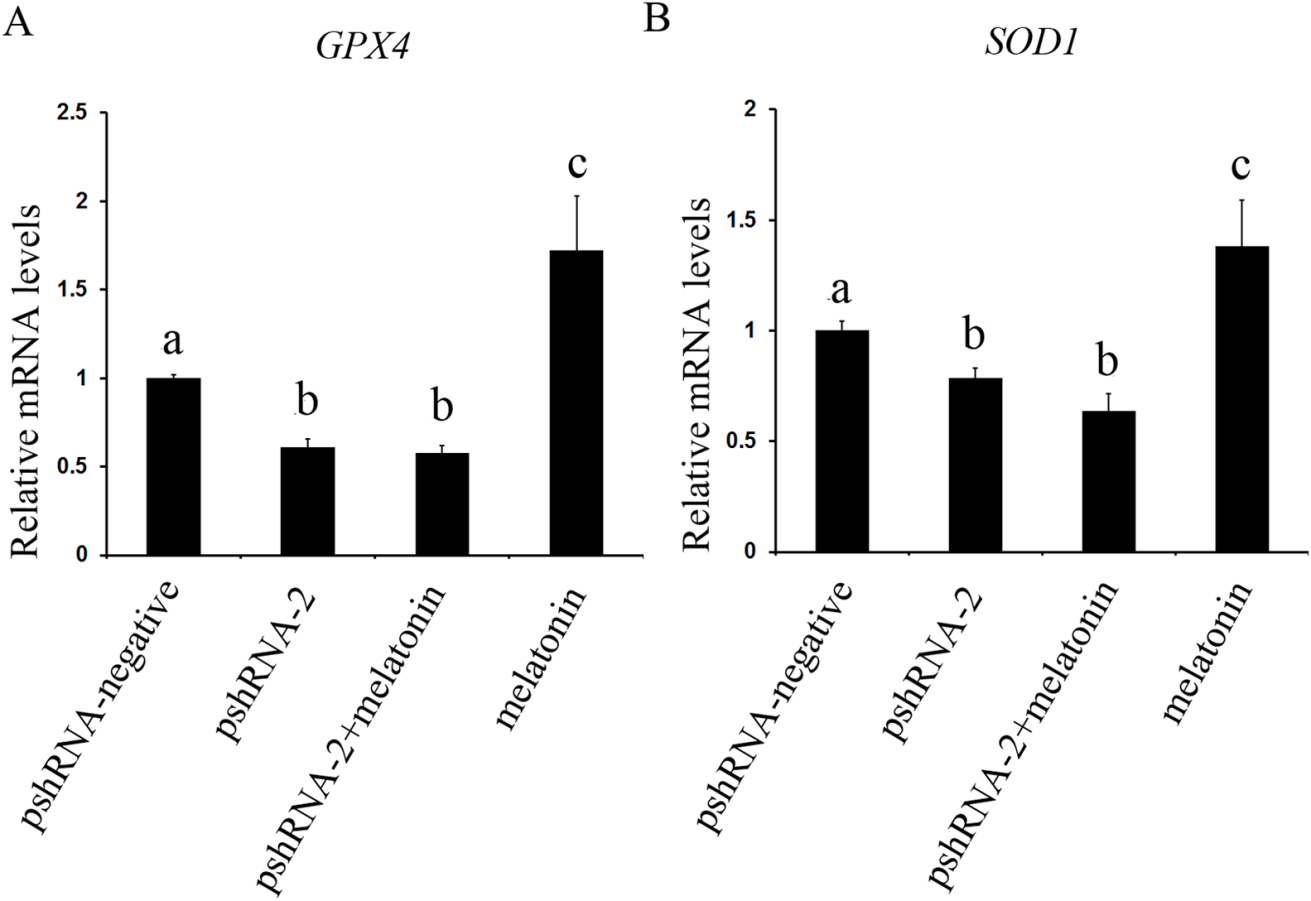
Effects of *MTNR1B* gene silencing and melatonin supplementation on expression of *SOD1* and *GPX4*. The mRNA levels of *SOD1* and *GPX4* were examined by real-time PCR in GCs at 48 h after transfection with pshRNA-3 and/or melatonin treatment. (A and B): The expression of *SOD1* and *GPX4* both were significantly downregulated in the pshRNA-2 group and pshRNA-2 + melatonin group compared to control group and there was no significant difference between the pshRNA-2 group and the pshRNA-2 + melatonin group. Moreover, melatonin significantly promoted the expression of *SOD1* and *GPX4* in the melatonin group compared to other groups. The quantity of mRNA was normalized to that of *ACTB*. The data with different lowercase letters (a, b, and c) were significantly different (*P* < 0.05).

The effect of *MTNR1B* silencing and melatonin treatment on the expression of *SOD1* and *GPX4* was assessed in bovine GCs. The expression of *SOD1* and *GPX4* both were significantly downregulated in the pshRNA-2 group and the pshRNA-2 + melatonin group compared to control group (Fig. 7, *P* < 0.05) and there was no significant difference between the pshRNA-2 group and the pshRNA-2 + melatonin group (*P* > 0.05). Moreover, melatonin significantly promoted the expression of *SOD1* and *GPX4* in the melatonin group compared to other groups (*P* < 0.05). Therefore, melatonin suppressed oxidative stress in GCs through upregulating the expression of *SOD1* and *GPX4*, and *MTNR1B* knockdown could affect melatonin regulating the expression of *SOD1* and *GPX4*.

## Discussion

Melatonin plays an important role in maintaining of mitochondrial homeostasis and protecting the integrity and function of cells, which depend on the anti-oxidant, anti-apoptosis, and free radical scavenging activity. However, here we tried to find out the role of melatonin receptor MTNR1B with regard to bovine GCs cell cycle, apoptosis, and anti-oxidant which was not investigated before, especially MTNR1B mediating the effects of melatonin on bovine GCs. Therefore, we investigated the effects of *MTNR1B* gene silencing on the apoptosis, cell cycle, and oxidative stress in the bovine GCs. Moreover, we further revealed whether *MTNR1B* silencing affected the effects of melatonin on GCs. The results indicated that *MTNR1B* RNAi vectors were designed and constructed, and stably transfected into bovine GCs, where they were expressed normally in the cells. *MTNR1B* mRNA and protein expression were significantly inhibited in bovine GCs. The pshRNA-2 plasmid was the most effective in silencing *MTNR1B* mRNA and protein expression. The effectiveness of this plasmid in cultured bovine GCs points to a potential in vitro approach to studying the mechanism by which melatonin and *MTNR1B* regulate the development and function of GCs.

It is well established that the cross talk between cell apoptosis and survival signals is crucial for follicular development, and cell proliferation, differentiation or apoptosis contribute to follicle ultimately ovulates or undergoes atresia (Matsuda et al., 2012; Choi et al., 2011; Kadariya et al., 2015). The apoptosis of GCs play an important role in the initiation of follicular atresia (Tilly et al., 1991; Jiang et al., 2003; Choi et al., 2011). Therefore, GCs are critical for follicular growth and atresia, maturation of oocytes, the estrous cycle and pregnancy maintenance after ovulation (Choi et al., 2011; Li & Albertini, 2013; Arosh et al., 2004). In the present study, *MTNR1B* knockdown significantly induced GCs apoptosis; in contrast, melatonin significantly suppressed the GCs apoptosis. Moreover, *MTNR1B* knockdown inducing the GCs apoptosis was reversed by melatonin treatment. Consistent with these, *MTNR1B* knockdown significantly suppressed the expression of *BCL2* and *BCL-XL*, while promoting the expression of *CASP3* and *BAX*, without significant alteration in the expression of *TP53*. In contrast, melatonin significantly promoted the expression of *BCL2* and *BCL-XL*, while suppressing the expression of *CASP3*, *TP53*, and *BAX*. Curiously, melatonin reversed the effects of *MTNR1B* knockdown on suppressing *BCL2* and promoting *CASP3* and *BAX* expression, while *MTNR1B* knockdown did not affect melatonin regulating the expression of *BCL2*, *BAX*, *TP53*, and *CASP3* except *BCL-XL* in GCs. These results were consistent with our previous reports that exogenous melatonin could suppress apoptosis in bovine GCs (Wang et al., 2012a) and melatonin could protect GCs against thermal stress-inducing apoptosis in sheep (Fu et al., 2014). Previous studies have revealed that melatonin plays an important role in maintaining optimal mitochondrial function and homeostasis and protecting the integrity and function of cells by protecting against mitochondrial oxidative stress, thus decreasing subsequent cell apoptosis (Tan et al., 2013; Jou et al., 2010). The BCL2 family take part in the mitochondrion-mediated apoptotic pathway, govern outer membrane permeabilization of mitochondria and are regarded as key factors in modulating apoptosis in female germ cells (Radogna et al., 2015; Espino et al., 2010; Kim & Tilly, 2004). Overexpression of *BAX* accelerates the cell apoptosis (Oltvai, Milliman & Korsmeyer, 1993) and the effect of *BCL2* overexpression, however, is completely reciprocal to that of *BAX* in mice growing follicles (Hsu et al., 1996). Radogna et al., (2007, 2008, 2015) indicated that melatonin suppressed cell propensity to apoptosis through promoting relocalization of the BCL2 to mitochondria and BCL2 sequestering BAX into mitochondria in an inactive form. In addition, the imbalance of BCL2/BAX inducing mitochondrial-mediated GCs apoptosis has been reported in mice (Kadariya et al., 2015), bovine (Chong et al., 2015; Wang et al., 2017a), goose (Wang et al., 2012b), and porcine (Zhen et al., 2014). CASP3 is an important effector molecule in promoting apoptosis in all types of cells, which initates cascade of caspases to execution of cells apoptosis (Han et al., 2013; Cai et al., 2011); the activation of CASP3 is suppressed after melatonin treatment and then modulates the CASP3-dependent apoptotic pathway (Baydas et al., 2005). TP53 is another important regulator of cell apoptosis, which activates mitochondrial caspases mediated apoptotic pathway (Chowdhury, Tharakan & Bhat, 2006; Brown et al., 2007). Melatonin mediates apoptosis related genes (*BCL2*, *TP53*, *CASP3*, and *BAX*) in porcine GCs predominantly through the activation of MTNR1B (He et al., 2016b). These findings confirm that *MTNR1B* knockdown increased the apoptosis in bovine GCs and melatonin inhibited GCs apoptosis via regulating pro- and anti-apoptotic related genes. Moreover, the role of *MTNR1B* knockdown in bovine GCs apoptosis was reversed after melatonin treatment, which suggested that *MTNR1B* knockdown did not affect melatonin response in bovine GCs.

CCND1 and CCNE1 are the key regulators of the cell cycle, which promote the progression from phase G1 to S (Zhen et al., 2014; M’baye et al., 2015; Alao et al., 2006; Ohtsubo et al., 1995). Whereas, CDKN1A is an important regulator that inhibits the restriction point from phase G1 to S (Han et al., 2013; Wang et al., 2012a; Harper et al., 1993). G1 phase arrest is induced in human osteoblastic cell after melatonin treatment (Liu et al., 2011). During ovulation, GCs undergoing luteinization lose the ability to divide and are arrested at G0/G1 (Green et al., 2000). In the present study, *MTNR1B* knockdown did not alter the cell cycle; however, melatonin increased the G1 phase of cell cycle accompanied with the decreased S phase. Consistent with this, melatonin decreased *CCND1* and *CCNE1* expression while increasing *CDKN1A* expression. Taken together, these results indicate that melatonin is important in modulating the cellular progression, particularly, GCs undergoing luteinization transformed into luteal tissue after ovulation. However, *MTNR1B* knockdown did not affect the cell cycle and the GCs respond to melatonin on regulating the GCs cycle.

Under physiological conditions, ROS is involved in multiple physiological processes of animal reproduction through acting as second messenger (Agarwal, Gupta & Sharma, 2005). However, the excessive ROS would surely damage structures and functions of cell. Therefore, balance must be maintained in ovary to ensure successful reproduction (Tamura et al., 2013). The presence of melatonin, a proven anti-oxidant, in the ovary likely helps to maintain this balance (Reiter et al., 2014). In addition, melatonin also prevents ROS from damaging the GCs, which is essential for the survival of GCs, follicular growth, the estrous cycle and pregnancy maintenance after ovulation (Matsuda et al., 2012; Choi et al., 2011; Arosh et al., 2004; Cruz et al., 2014a). In the present study, melatonin improved bovine GCs oxidative capacity by increasing the expression of anti-oxidant enzymes *SOD1* and *GPX4* genes. *MTNR1B* knockdown decreased the expression of *SOD1* and *GPX4*. Moreover, *MTNR1B* knockdown could fully inhibit the increasing of *SOD1* and *GPX4* invoked by melatonin. These results were consistent with previous reports that the expressions of *GPX4* and *SOD1* were increased after melatonin supplementation in bovine embryo and porcine oocyte (Wang et al., 2014; Li et al., 2015). An additional positive effect of melatonin on porcine GCs appears to be through improving the genes expression of encoding the anti-oxidant enzymes *SOD1* and *GPX4* (He et al., 2016a). Melatonin protects the integrity of preovulatory follicle GCs by inducing the expression of anti-oxidant enzymes, reducing oxidative stress in the mouse GCs and maintaining of mitochondrial homeostasis (Tanabe et al., 2015; He et al., 2016a; Cruz et al., 2014b). Recent work reveals that melatonin is able to protect mice against stroke by activating MTNR1B receptor, which reduces oxidative stress (Chern et al., 2012). In another study, melatonin mediates anti-oxidant enzymes *SOD1* and *GPX4* genes in porcine GCs predominantly through the activation of MTNR1B (He et al., 2016b).

Although MTNR1A and MTNR1B show 70% sequence identity at membrane domains, and 55% identity at the amino acid level (Reppert, Weaver & Ebisawa, 1994; Reppert et al., 1995), it is considered that MTNR1A and MTNR1B play distinct roles. For example, it has been demonstrated MTNR1A is critical for normal brain function in MTNR1A knockout mice (Weil et al., 2006), and plays important role in photoperiodic regulation of gonadal activity (Yasuo et al., 2009). MTNR1B modulates immune and inflammatory responses, as well as the behavioral effect in knockout mice (Drazen & Nelson, 2001; Drazen et al., 2001; Larson et al., 2006). However, MTNR1A and MTNR1B is reported to mediate the regulation of complex reproductive mechanisms (Dubocovich & Markowska, 2005); for instance, MTNR1A mediates melatonin controling behavior and reproductive function in a seasonal reproductive mammal (Prendergast, 2010), as well as melatonin and MTNR1A are involved in the downstream of human chorionic gonadotropin stimulation and play important role in luteinization (He et al., 2016d). On the other hand, the role of melatonin in regulating porcine GCs proliferation and apoptosis are predominantly mediated by MTNR1B (He et al., 2016c). Melatonin increases serum estradiol and decreases ovarian Gonadotropin-inhibitory hormone receptor expression, thus improves hens egg-laying rates by activation of MTNR1B (Jia et al., 2016). However, MTNR1A and MTNR1B may act in a complementary way to modulate the cell apoptosis. Evidence for such complementary role includes the follow: the anti-apoptotic effects of melatonin on spermatozoa is mediated by activation of MTNR1A and/or MTNR1B receptors (Espino et al., 2011); further, melatonin modulates the cell life/death balance through interaction with the MTNR1A and MTNR1B (Radogna et al., 2008); Similarly, MTNR1A and MTNR1B, which are under the positive control of melatonin, modulate the cell life/death balance of human leucocytes (Espino, Rodríguez & Pariente, 2013). In addition, our recent research indicates that melatonin and MTNR1A play an important role in modulating bovine GCs function by regulating cellular progression and apoptosis. Furthermore, *MTNR1A* silencing could not block bovine GCs respond to melatonin (Wang et al., 2017b). Consistent with this, *MTNR1B* silencing did not disrupting the effects of melatonin on apoptosis and cell cycle in bovine GCs and melatonin enhanced the expression of *MTNR1A* with or without *MTNR1B* knockdown in the present research. Therefore, MTNR1A and MTNR1B may work in concert to modulate bovine GCs function by regulating cellular progression and apoptosis.

## Conclusion

The present results disclose that melatonin and MTNR1B have an important role in regulating the GCs apoptosis, cell cycle, and oxidative stress. *MTNR1B* knockdown significantly promoted the GCs apoptosis, while melatonin significantly suppressed the GCs apoptosis. Moreover, *MTNR1B* knockdown did not disrupt the effect of melatonin on GCs apoptosis and cell cycle. Accumulating evidence suggests the beneficial role of melatonin and MTNR1B in regulating apoptosis, oxidative stress, and cell cycle progression in GCs is essential for folliculogenesis in the bovine.

## Supplemental Information

10.7717/peerj.4463/supp-1 Supplemental Information 1 Effects of *MTNR1B* gene knockdown and melatonin supplementation on related genes expression, cell cycle and GCs apoptosis. Raw data for Ct value of 11 target genes and 1 reference gene, cell cycle and cell apoptosis in four experimental group: pshRNA-negative group (control), pshRNA-2 group, pshRNA-2 plus melatonin group and melatonin group.

10.7717/peerj.4463/supp-2 Supplemental Information 2 Supplemental Figures S1–S7. MTNR1B, BCL2, BAX, CASP3, TP53 and ACTB in the pshRNA-negative group, pshRNA-2 group, pshRNA-2 plus melatonin group, and melatonin group detected by the Western blot.

## Additional Information and Declarations

### Data Availability

The following information was supplied regarding data availability: The raw data has been supplied as a Supplementary Dataset File.
