# Supplementary figures and images for: RNAi-mediated knockdown of MTNR1B without disrupting the effects of melatonin on apoptosis and cell cycle in bovine granulose cells
Source: PeerJ. 2018 Apr 23;6:e4463. doi: 10.7717/peerj.4463 (PMC5918132; doi:10.7717/peerj.4463)

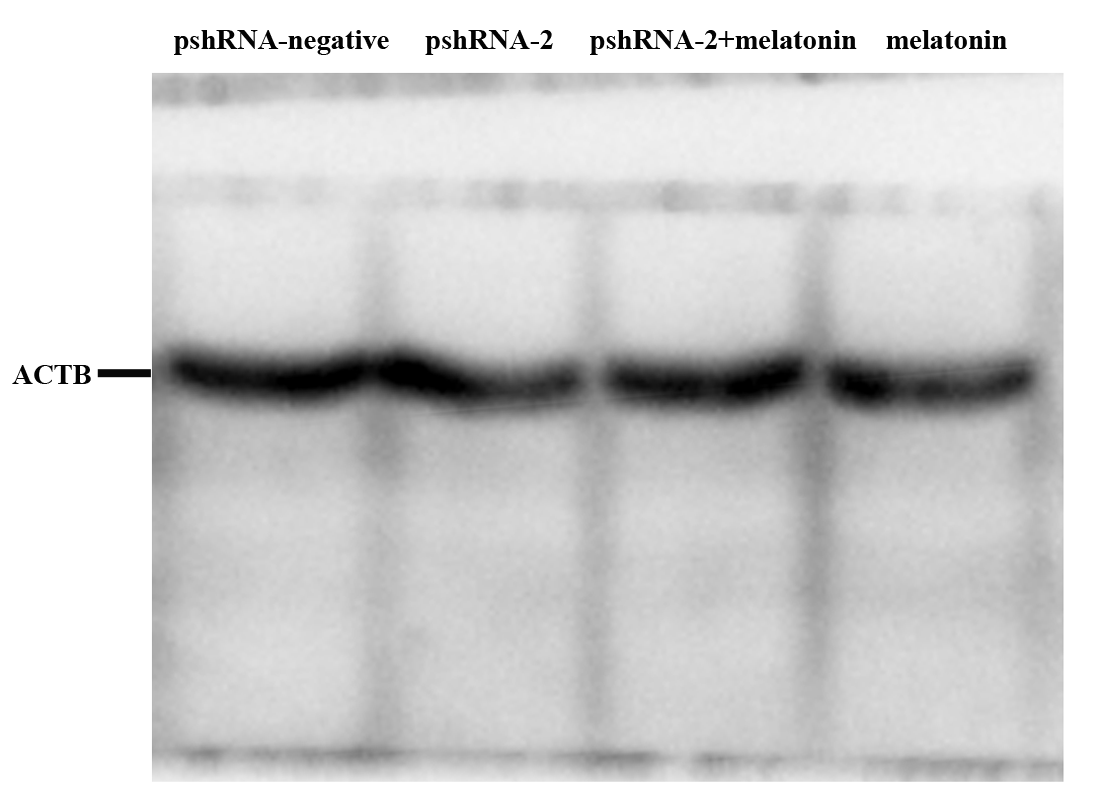

Supplement: Supplemental Information 2 — MTNR1B, BCL2, BAX, CASP3, TP53 and ACTB in the pshRNA-negative group, pshRNA-2 group, pshRNA-2 plus melatonin group, and melatonin group detected by the Western blot. [file peerj-06-4463-s002.zip › Supplemental ACTB S2.png]

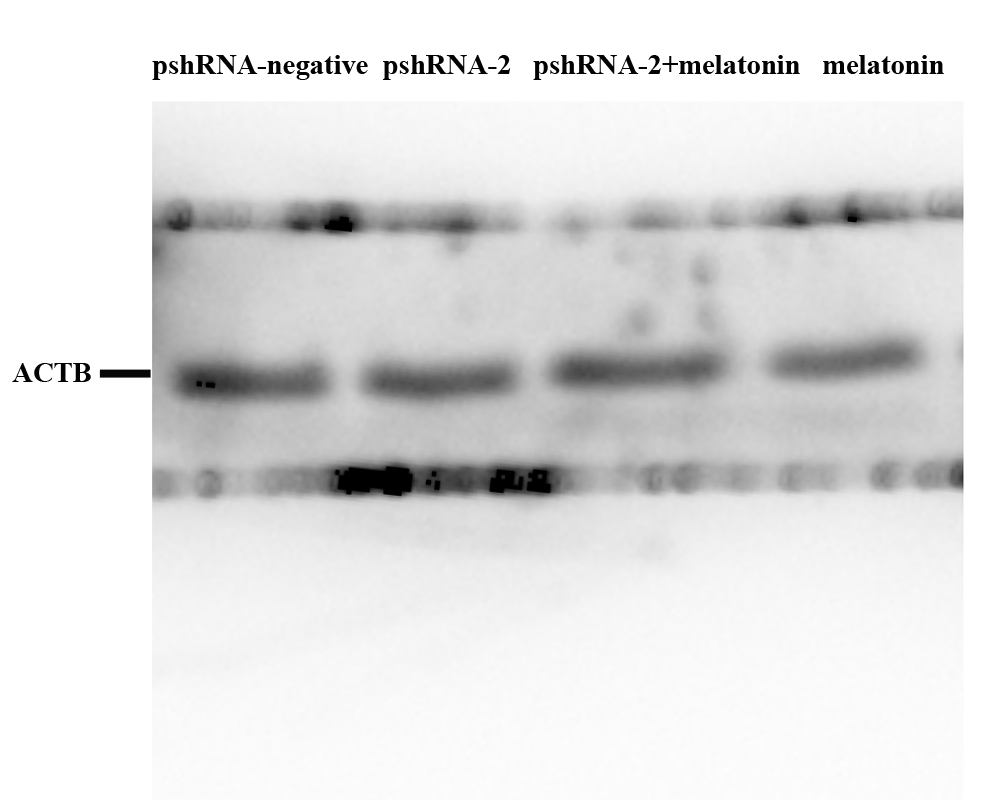

Supplement: Supplemental Information 2 — MTNR1B, BCL2, BAX, CASP3, TP53 and ACTB in the pshRNA-negative group, pshRNA-2 group, pshRNA-2 plus melatonin group, and melatonin group detected by the Western blot. [file peerj-06-4463-s002.zip › Supplemental ACTB S7.png]

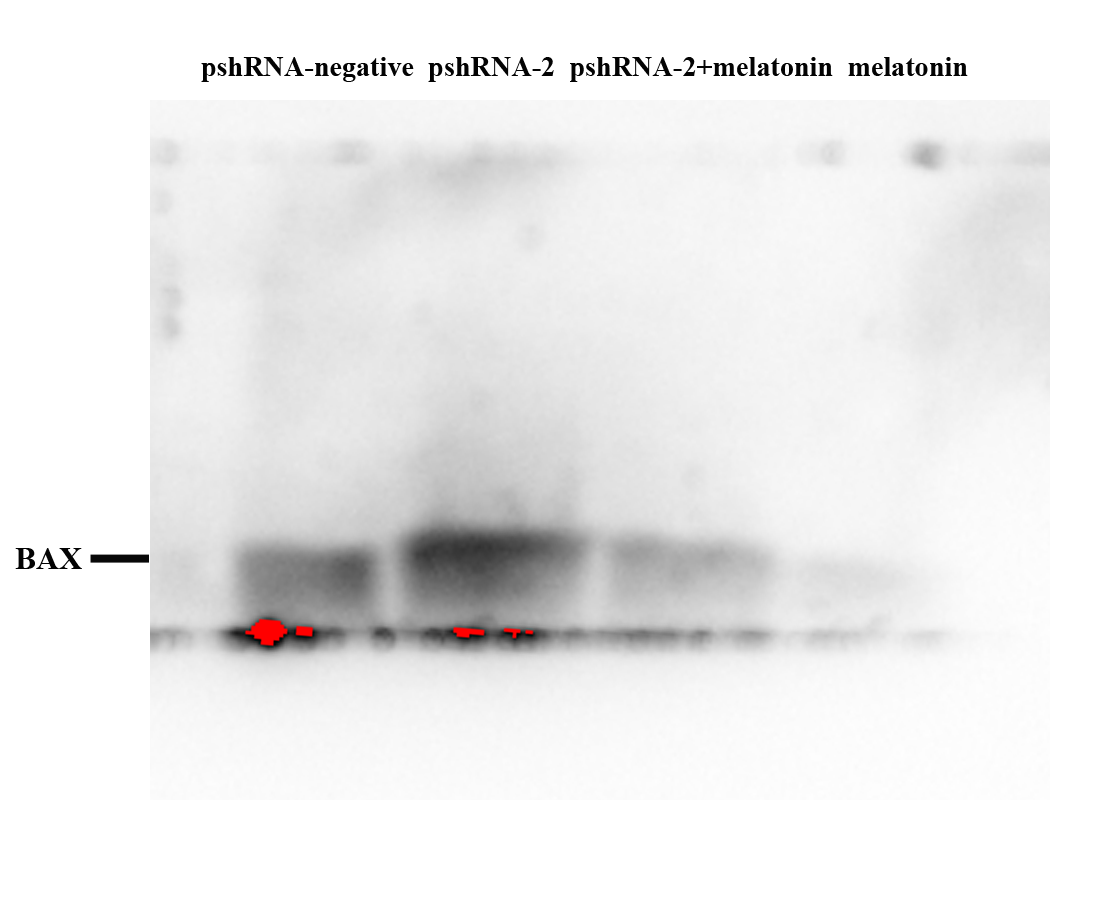

Supplement: Supplemental Information 2 — MTNR1B, BCL2, BAX, CASP3, TP53 and ACTB in the pshRNA-negative group, pshRNA-2 group, pshRNA-2 plus melatonin group, and melatonin group detected by the Western blot. [file peerj-06-4463-s002.zip › Supplemental BAX S4.png]

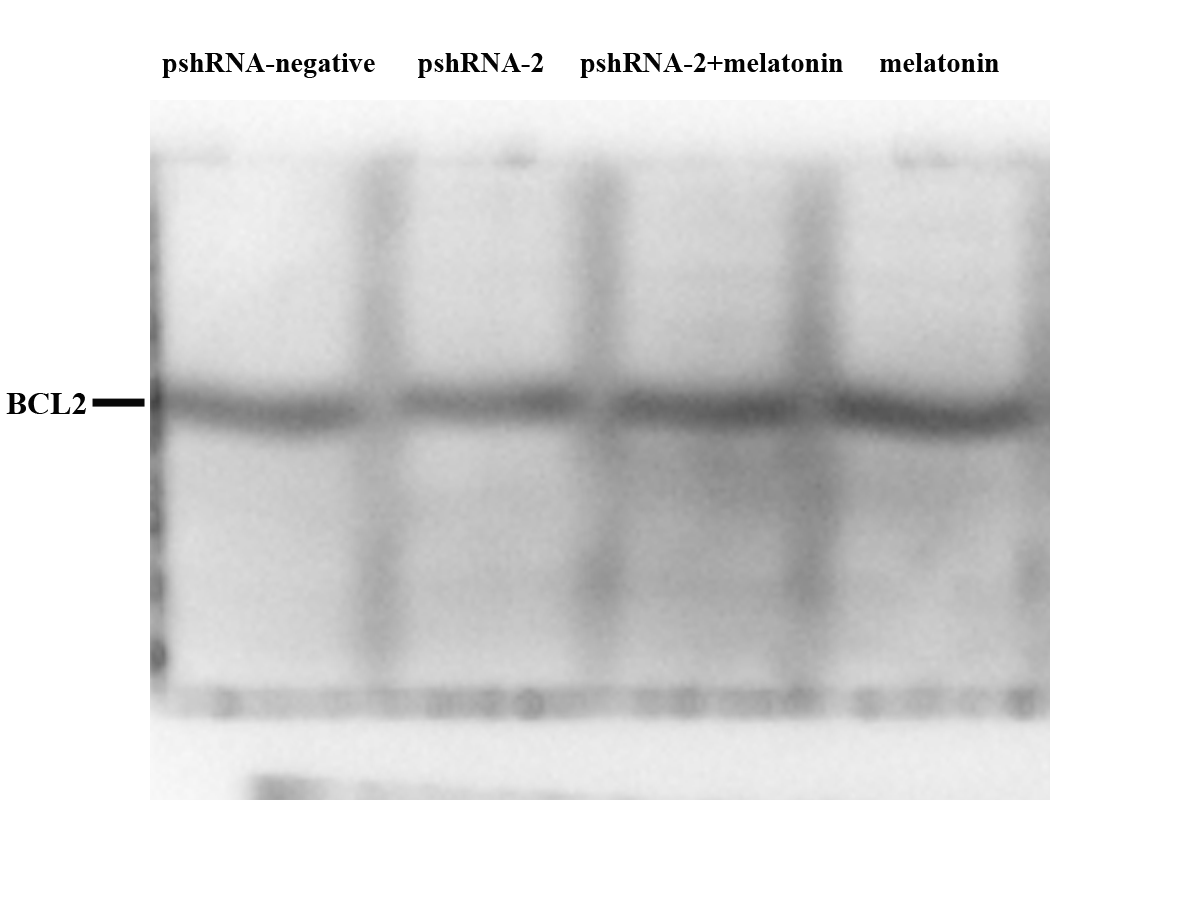

Supplement: Supplemental Information 2 — MTNR1B, BCL2, BAX, CASP3, TP53 and ACTB in the pshRNA-negative group, pshRNA-2 group, pshRNA-2 plus melatonin group, and melatonin group detected by the Western blot. [file peerj-06-4463-s002.zip › Supplemental BCL2 S3.png]

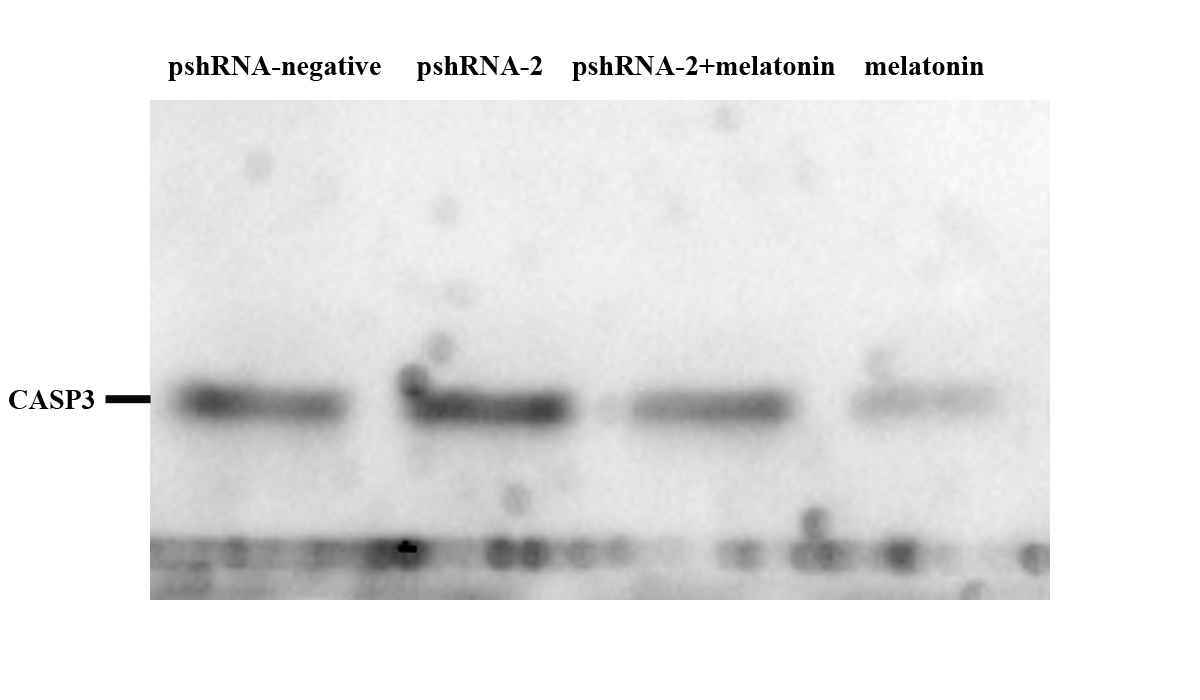

Supplement: Supplemental Information 2 — MTNR1B, BCL2, BAX, CASP3, TP53 and ACTB in the pshRNA-negative group, pshRNA-2 group, pshRNA-2 plus melatonin group, and melatonin group detected by the Western blot. [file peerj-06-4463-s002.zip › Supplemental CASP3 S5.png]

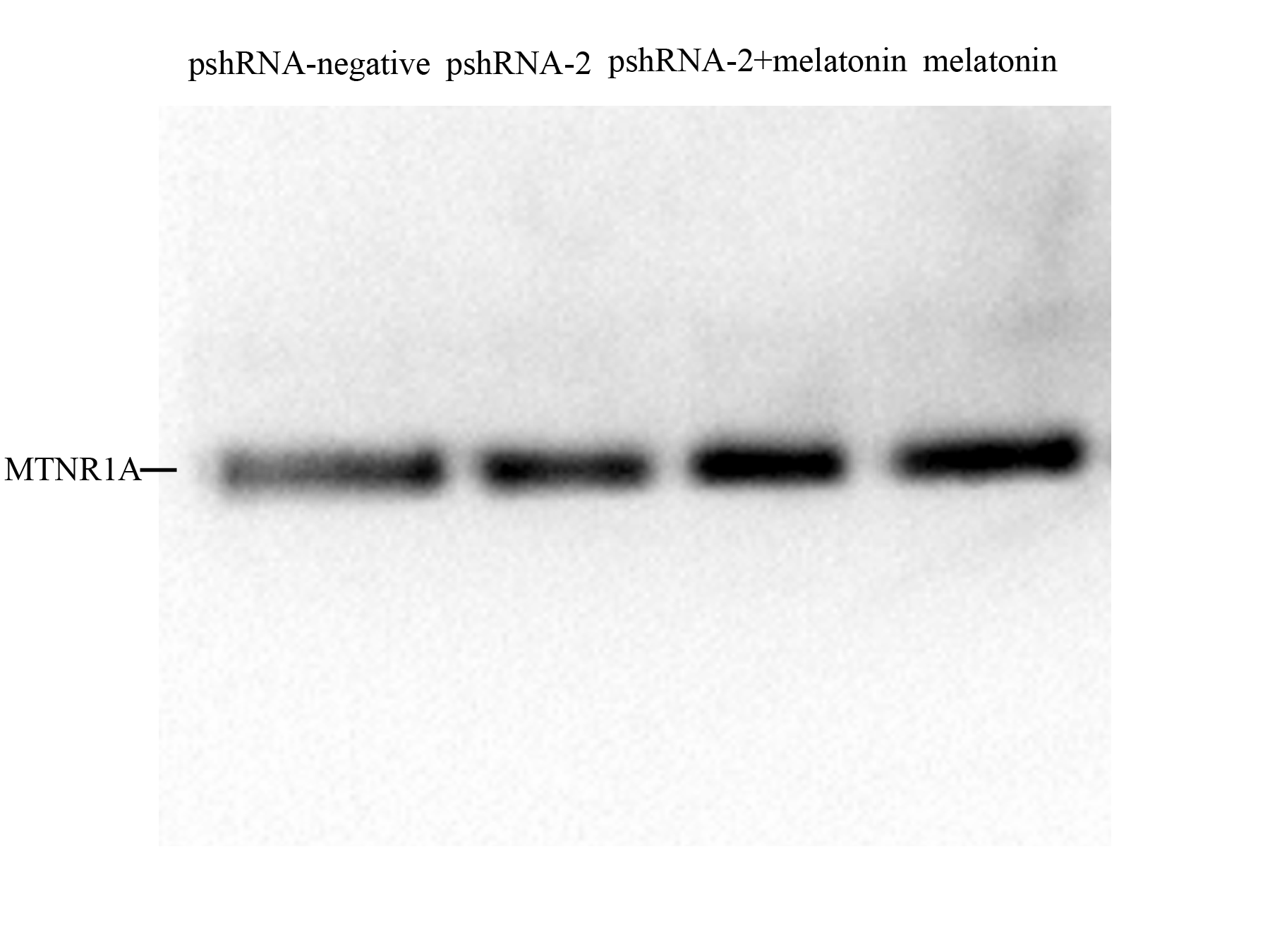

Supplement: Supplemental Information 2 — MTNR1B, BCL2, BAX, CASP3, TP53 and ACTB in the pshRNA-negative group, pshRNA-2 group, pshRNA-2 plus melatonin group, and melatonin group detected by the Western blot. [file peerj-06-4463-s002.zip › Supplemental MTNR1A S8.png]

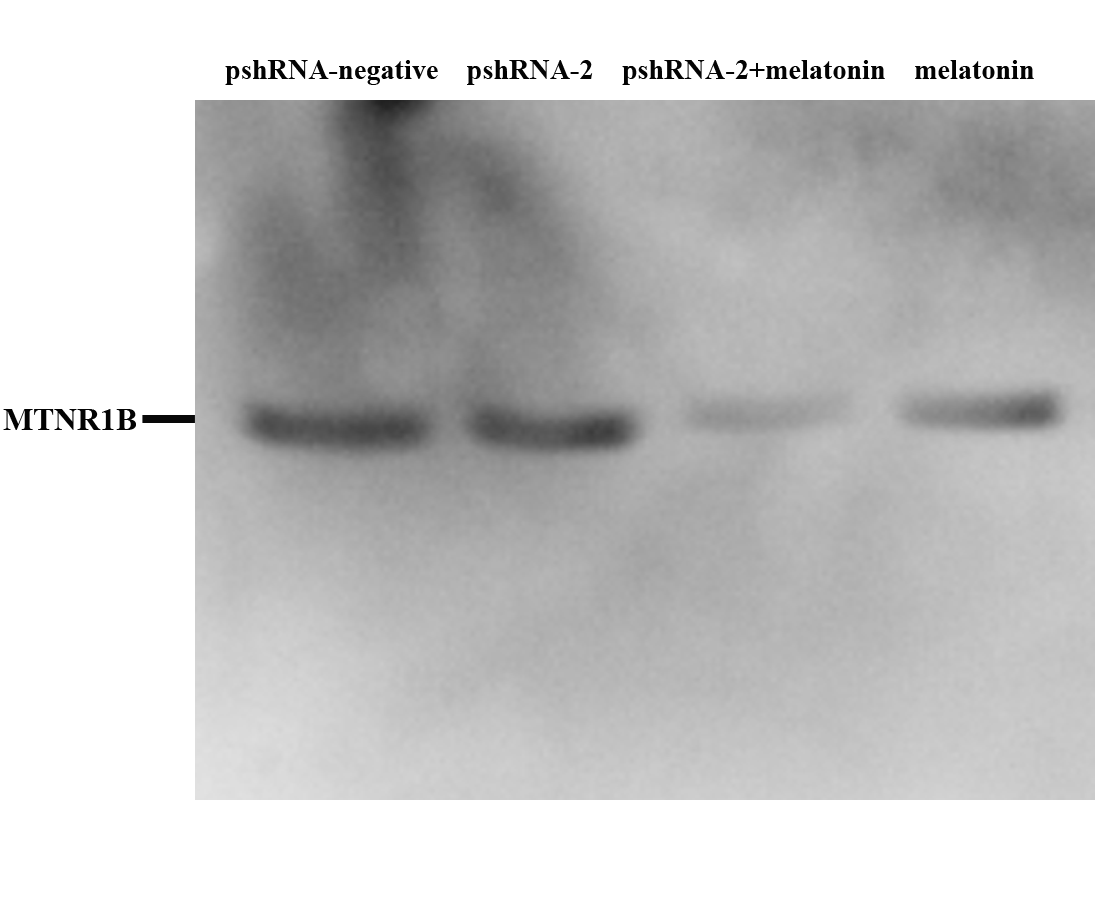

Supplement: Supplemental Information 2 — MTNR1B, BCL2, BAX, CASP3, TP53 and ACTB in the pshRNA-negative group, pshRNA-2 group, pshRNA-2 plus melatonin group, and melatonin group detected by the Western blot. [file peerj-06-4463-s002.zip › Supplemental MTNR1B S1.png]

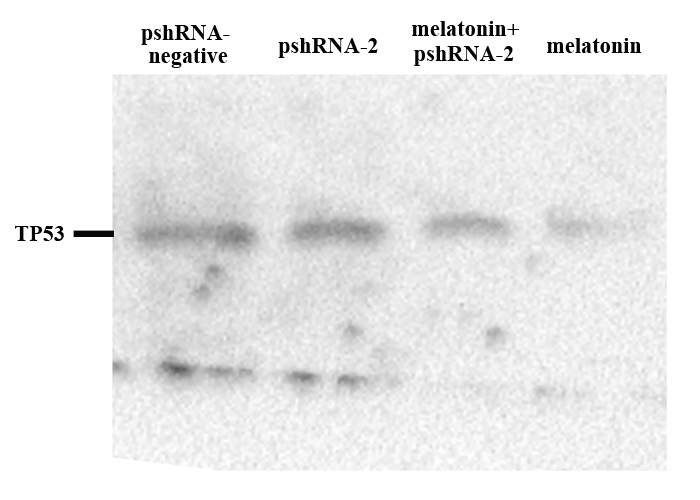

Supplement: Supplemental Information 2 — MTNR1B, BCL2, BAX, CASP3, TP53 and ACTB in the pshRNA-negative group, pshRNA-2 group, pshRNA-2 plus melatonin group, and melatonin group detected by the Western blot. [file peerj-06-4463-s002.zip › Supplemental TP53 S6.png]
